# The Prevention of Diarrhea, with Special Reference to Bacteriology

**Published:** 1904-08

**Authors:** Robt. W. Hynds

**Affiliations:** Atlanta, Ga.; Assistant Bacteriologist Atlanta Board of Health


### THE PREVENTION OF DIARRHEA, WITH SPECIAL REFERENCE TO BACTERIOLOGY.*

By ROBT. W. HYNDS, M.D.,

Assistant Bacteriologist Atlanta Board of Health.

The etiology of diarrheal diseases in children falls under three general headings.

1. Poisoning from toxines formed in the food before it is ingested.

2. Infection from without, or infection introduced with the food.

3. Infection from within, or those conditions in which the

*Read before Atlanta Society of Medicine, July 21, 1904.

micro-organisms usually found in the intestine assume pathogenic properties.

As the statistics show that only three per cent. of the fatal cases of diarrhea occur in breast-fed infants we may assume that food infection is the main factor in the production of these troubles. This being true and as cow's milk is the most frequent substitute for mother's milk the problem of securing a pure milk supply assumes great importance, and it is toward that phase of the question that most of this paper will be directed.

It must be remembered, however, that a considerable percentage of cases occur in infants fed upon condensed milk and proprietary foods. In this class of cases we must look for infection from the water, unclean nursing-bottles, etc., and the increased difficulty of digestion of these foods lessens the resisting power of the intestinal canal. Condensed milk left open for some time in a warm place may become contaminated.

Vaughn first called attention to a violent type of gastrointestinal trouble due to the formation of a poisonous toxin in the milk. He succeeded in isolating the toxin and called it tyrotoxin Trimethylamine, likewise a product of putrefaction, was isolated from milk by Bruger, and there are probably similar compounds that give rise to diarrhea.

Much has been written and many conflicting opinions advanced regarding the micro-organism, or micro-organisms, said to produce summer diarrhea and dysentery. The claim of those observers who regard the bacillus dysenteriæ of Shiga as the specific cause of summer diarrhea and dysentery are founded on insufficient evidence, and are not borne out by the facts. That the bacillus, first described by Shiga in 1897, and more fully by Kruse in 1901, is a bacteriological entity, and not merely a modification of the colon bacillus has not been fully demonstrated, and cultures of this bacillus fail to produce diarrhea in humans.

Maggiora, Laveran, Arnaud, Celli, Fiocca and Nowak all attribute dysentery and other types of diarrheal disease to the bacillus coli. That the colon bacillus under certain conditions secretes toxic compounds in the intestinal canal which produce diarrhea can not be doubted. Robert has isolated a poison from the stools in this class of cases which produce a violent diarrhea when injected in rabbits. Of those diarrheas arising from infection from within the greater number are probably due to the bacillus coli. This micro-organism, normally benign, but capable of undergoing so many modifications, may under certain conditions assume pathogenic properties and give rise to diarrhea.

Ziegler, Babes, Ogata and Roger all described special bacilli, each claiming to have found the specific cause of dysentery. The bacilli enteritides sporogenes of Klein was held by Klein, Andrews and others to be the cause of cholera infantum. Silvestre described a diplococcus which he claimed produced dysentery. A similar claim was advanced by Hiss and Russell for the bacillus Y. The streptococcus, proteus vulgaris and staphylococcus have all been named as the exciting factor. According to Escherich and others the main causative agent in infection from without the intestine is the streptococcus. This claim is substantiated by Blum and others, and that the streptococcus may produce a violent enteritis is beyond a doubt.

Jurgens disputes the assertion that a bacillus of the Shiga-Kruse type is the only cause of summer diarrhea, and says that it is due to a mixed infection. This is probably the true solution of the origin of some cases, as we know that two or more varieties of bacteria which are perfectly harmless when taken separately may become pathogenic when mixed.

As predisposing causes to infection from within the intestine we have a lowered resistance of the patient, nature of the intestinal contents and condition of the mucous membrane. The ptomaines formed by the action of the putrefactive bacilli diminish the resistance of the intestinal mucous membrane, and any disturbance of the digestive organs seem to increase the virulence of the micro-organisms colonized in the intestinal canal. Any excessive growth of bacteria in the intestines, no matter of what type, injures the body, for Professor Bouchard called attention to the fact that the feces possessed a toxic character, containing a variety of alkaloids and an increased bacterial growth leads to an increased production of these.

In view of the fact that so large a percentage of cases of diarrhea occur in infants fed upon cow's milk, it might be well to consider briefly the bacteriology of cow's milk. Conn has listed over two hundred species of bacteria found in cow's milk, very few of which seem to possess any significance in the production of diarrheal diseases. Park and Hall in a large series of investigations isolated 135 species which failed to produce any ill effects on kittens. They state, however, that a million bacteria to the cubic centimeter is certainly deleterious.

The two main groups of bacteria usually found in milk are the lactic acid producing group, and the putrefactive or peptonizing bacilli. The former of these is derived from accidental contamination, never being found in the milk-ducts. They are exceedingly useful in inhibiting the growth of the putrefactive bacilli. The lactic acid producing group includes over one hundred varieties. Only two are of any special importance: A facultative anaerobic and an aerobic type. Members of the former group have been given a great variety of names, but are probably best classed under the name of bacterium lactis acidi. The aerobic type are normally present only in small numbers, and includes the bacillus coli and the various bacilli said to produce dysentery described by Shiga and others.

The bacillus pyocyaneus, bacillus proteus vulgaris and varities of the staphylococcus are normally found in milk. Several varieties of the streptococcus appear so constantly in milk that they may be considered as being normally present in the milk-ducts. Only in cases of mammitis do they appear to give rise to trouble. Sometimes micro-organism are present that produce changes that can be readily detected by the eye, as the bacilli producing blue milk, ropy milk and alkaline fermentation.

Although summer diarrheas are mainly due to food infection, yet there are several predisposing or contributing causes we must take cognizance of in seeking to prevent them. Probably the most important of these are impure water and a lack of fresh air. The municipalities should provide a sufficient number of parks, convenient of access, where the infants and children may escape from the stale, dust-laden air of the crowded districts.

Overfeeding is a frequent contributing cause of summer diarrhea. It is sometimes necessary to decrease the quantity of food, and is often desirable to decrease the percentage of solids in the hot season. A reduction in the percentage of fat during the season will often prevent an attack of intestinal indigestion. During the summer the absorptive powers of the infant are frequently lowered, and as a result of overfeeding may have undigested milk remaining in the intestines, which provides an ideal culture medium for bacteria. Another source of infection which must be considered is the common house-fly, by which the infective agent may be carried to the feeding-bottle or hand of the infant.

A phase of the question which has been much neglected is the disinfection of the feces. It is as important to disinfect the feces in dysentery as in typhoid.

Since the establishment of the fact that a modified cows milk is the best substitute for mother's milk the problem of securing a pure milk supply has assumed vital importance in the prevention of diarrhea, and pure-milk crusades have sprung up all over the country. The mortality of infants in summer, fed upon condensed milk is much greater than among those fed upon pure cow's milk.

Behring contends that the large infant mortality of our cities is due to the use of heated milk. He claims that fresh milk contains a powerful antibody against the colon bacillus, which is destroyed by heat. He recommends the feeding of pure, raw cow's milk, and where this can not be obtained the feeding of milk preserved with formalin. According to the experiments of Jensen calves fed boiled milk developed a violent diarrhea similar to calf dysentery.

Contamination of the milk may take place at the dairy, in the handling of the product and in the home. According to Conn the number of bacteria in a cleanly kept dairy varies from one to ten thousand per cubic centimeter. Under poor conditions the number often rises to three or four hundred thousand. One sample containing three thousand per c. c. kept by Park at 86 degrees for twenty-four hours went to 1,400,000,000 per c. c. At 50 degrees it showed no increase whatever. Milk can not be obtained absolutely free from bacteria, as they are found normally in the milk-ducts. They obtain entrance to the ducts from external sources, and are not normally present in large numbers, and very rarely above the milk cistern. In cows with diseased udders the ducts contain large numbers of streptococci, and this form of infection is frequent.

The admission of preventable dirt is the main source of infection at the dairy. Particles of excrement may be dislodged from the udder through the manipulation attendant upon milking and fall into the pail. Bacteria-laden hair may be shaken into the milk. The switching of the cow's tail scatters a shower of dirt, and her movements dislodge the dried manure on her unclean flanks. Harrington says that half a grain of such dirt will produce three and a half million of bacteria per c. c. by the time it is delivered. Russell collected on a gelatin plate the size of an ordinary pail, exposed under a cow during the milking for one minute, some six thousand bacteria. The air of the barn is ordinarily filled with bacteria-laden dust, especially when dry food is given to the cows just before or during milking. Other sources of contaminatian at the dairy are the milk-vessels and the person of the milker. Dirty hands, especially if the process of wet-milking is used, give rise to dirty milk. A sterile milk pail, according to Russell, gave rise to only 165 bacteria per c. c., while one cleaned the ordinary way yielded over 4,000.

The second source of contamination of milk mentioned was carelessness in the handling. The milk may be run over a dirty aerator in a dust-laden milk-room, and be received in unclean bottles or cans, or the bottles may be collected and refilled on the route without cleaning. In the summer the custom of leaving the bottle of milk for some time on the doorstep will often cause such a rise in the number of bacteria as to unfit it for infant feeding.

Carelessness in the handling of milk in the home, and improper cleansing of the nipple and feeding-bottle are responsible for many cases of diarrhea.

It is unnecessary here to enter at length into the means to be employed to produce a pure milk supply. Well-groomed cows, well-drained stables, with tight floors and ceilings, a minimum of 500 cubic feet of a air-space per cow, cleanly clothed milkers, sterilized vessels and the immediate removal of the milk to separate, clean milk-room are necessary. The most efficient means of reducing the number of bacteria in milk is immediate cooling. When kept at 50 degrees the number of bacteria do not increase, and at 45 degrees there is actually a decrease in their number. As soon as the milk is removed to the milk-room it should be poured into sterile bottles, sealed and kept at a low temperature until delivery, being subjected to as little manipulation as possible. Aeration is unnecessary as the cowy flavor of milk is due to an unclean barn.

Of the above points to be observed in the production of pure milk that of rapid cooling is the most important. Dirty milk if cooled rapidly will keep longer than clean milk not cooled. One point which I wish to emphasize is that too much dependence can not be placed in the pasteurization and sterilization, as these processes are ordinarily carried out in the home. They are often worse than useless, lending a false sense of security and causing one to overlook the dirty condition of milk.

How may the production of a sanitary milk supply be brought about? Through education of the producer, the distributor and consumer, together with proper supervision by the health authorities or a properly constituted milk commission. We must be able to offer to the dairyman a reward in the shape of higher prices for pure milk. We must teach the public the importance of pure milk and cause them to expect and demand it. As long as the public is indifferent, and seeks the cheapest and not the best milk, the dairymen will not strive to produce clean milk. It is your duty as physicians to educate the people and to impress upon them the necessity for clean milk. The fact that the milk is heated before it is fed will not always render it safe, as the heat can not destroy toxines already formed. This method is associated with the additional disadvantage of destroying certain chemical compounds, necessary to the proper nutrition of the infant. Besides this the sterilized milk is in itself a factor in the production of summer diarrhea, for the beneficent acid-producing bacteria having been destroyed the putrefactive bacilli hold free sway in the intestine.

We often hear it stated that it is impossible to produce pure milk. This is untrue, and if the milk is unclean it is not the fault of the cow but of some one connected with the dairy. Laws will do a great deal towards producing clean milk, but if the customer will examine the milk when delivered and if found unclean reject it, the milkman will stop offering dirty milk and the problem will be solved.

